# Comparing the efficacy of non-invasive physical therapy in improving pain and joint function of knee osteoarthritis: A protocol for systematic review and network meta-analysis

**DOI:** 10.1097/MD.0000000000025671

**Published:** 2021-05-07

**Authors:** Weisen Cai, Daoming Xu, Anju Xiao, Zongguang Tian, Tong Wang

**Affiliations:** a Department of Rehabilitation Medicine, Rehabilitation Hospital of Huishan Wuxi, Wuxi, Jiangsu Province; b Department of Acupuncture and Rehabilitation Medicine, Affiliated Hospital of Nanjing University of Chinese Medicine; c Department of Rehabilitation Medicine, The First Affiliated Hospital of Nanjing Medical University, Nanjing, China.

**Keywords:** knee osteoarthritis, network meta-analysis, physiotherapy, protocol

## Abstract

**Background:**

The incidence of knee osteoarthritis is increasing year by year, which seriously affects people's quality of life, especially the elderly, and has become a major public health problem. A lot of evidence shows that physical therapy has advantages in the treatment of knee joints, but there are a number of physical therapy schemes, and the efficacy of each scheme is different. This study will evaluate the clinical efficacy of different physical therapy regimens in the treatment of knee osteoarthritis (KOA) by means of network meta-analysis.

**Methods:**

According to the search strategy, we will retrieve the randomized controlled studies of non-invasive physical therapy in the treatment of knee osteoarthritis from CNKI, Wanfang, VIP, China Biomedical medicine, PubMed, Embase, Web of Science, and The Cochrane Library databases. The retrieval time was from the establishment of the database to March 2021. We will assess the quality of the studies using the Cochrane Risk Bias Assessment Tool and assess the strength of the evidence using the GRADE methodology. All data analyses will be performed by RevMan5.3, GEMTC 0.14.3, and Stata 14.0.

**Results:**

This study will evaluate the efficacy of different physical therapy in the treatment of knee osteoarthritis by evaluating the total response rate, pain relief degree, joint function score, quality of life score, adverse reactions, etc.

**Conclusions:**

This study will provide a reliable evidence-based basis for the selection of better physical therapy for the treatment of knee osteoarthritis.

**OSF Registration number:**

DOI 10.17605/OSF.IO/VX98B

## 1 Introduction

Knee osteoarthritis (KOA) is a common degenerative disease of knee joint characterized by cartilage degeneration, cartilage exfoliation, and subchondral hyperostosis. It could cause knee joint pain, joint instability and dysfunction, and seriously affects the quality of life of patients, which is a major public health problem.^[1,2]^ Knee osteoarthritis often occurs in the elderly, and the incidence is as high as 30% to 50% in people over 65 years old.^[3]^ It is one of the osteoarthritis that has the greatest impact on the quality of life of the elderly, and is also the main cause of lower limb disability.^[4]^ Osteoarthritis Research Society International (OARSI) recommends that the first-line management of knee osteoarthritis is still conservative treatment rather than surgery, emphasizing the importance of conservative treatment in the diagnosis and treatment of knee osteoarthritis.^[5]^ American College of Rheumatology (ACR) proposed that conservative treatment of KOA includes drug therapy and non-drug therapy, and non-drug therapy includes exercise and physical therapy.^[6]^ Drug therapy mainly includes analgesics, non-steroidal anti-inflammatory drugs, and corticosteroid injections. Although the above drugs have certain curative effects, they also have significant side effects.^[7,8]^

Physical therapy refers to the use of force (movement and pressure), sound, light, electricity, magnetic, cold, heat, and other physical methods to treat local or systemic dysfunction or lesions of the human body, in order to achieve the goals of anti-inflammation, pain relief, improvement of physical function, and so on.^[9]^ Physical therapy mainly includes movement therapy, modality therapy, and manual therapy, and is the most common treatment method for conservative treatment of osteoarthritis. Some clinical studies have shown that physical therapy can effectively improve the microcirculation around the joint, reduce the occurrence of secondary inflammatory reaction, delay the degeneration of cartilage,^[10]^ relieve symptoms in the short or long term, improve joint function, and reduce the need for painkillers.^[11–13]^ There are many kinds of physical therapy, and the effect and clinical efficacy are different. At present, there is no direct comparison of efficacy between different physiotherapy regimens. Therefore, we conducted this network meta-analysis to evaluate the efficacy and safety of different physical therapy regimens in the treatment of KOA, so as to provide comprehensive evidence for the selection of the optimal physical therapy regimens in the clinical treatment of KOA.

## 2 Methods

### 2.1 Protocol register

This protocol of systematic review and meta-analysis has been drafted under the guidance of the preferred reporting items for systematic reviews and meta-analyses protocols (PRISMA-P). Moreover, it has been registered on open science framework (OSF) (Registration number: DOI 10.17605/OSF.IO/VX98B).

### 2.2 Ethics

Since this is a protocol with no patient recruitment and personal information collection, the approval of the ethics committee is not required.

### 2.3 Eligibility criteria

(1) Type of studies: Randomized controlled trials (RCTs) are not limited to blind method and the language is limited to Chinese and English literature.
(2) Study control: For patients diagnosed with knee osteoarthritis, the diagnostic criteria are based on the KOA diagnostic criteria established by American College of Rheumatology (ACR) or Chinese Orthopaedic Association (COA),^[14]^ and sex, race, age, and occupation are not restricted.
(3) Intervention measures: the treatment group includes various physical therapy programs, including exercise therapy, muscle strength training, physical factor therapy, plan combination and others, while the control group is the comparison of drug therapy or other physical therapy.
(4) Exclusion criteria
  ① Secondary knee osteoarthritis: such as rheumatism, trauma and other secondary KOA, or combined with other arthritis;
  ② Studies which is unable to extract relevant data from published results, and unable to obtain original data after contacting the author;
  ③ Studies in which intervention measures are inconsistent with the research;
  ④ Studies published repeatedly;
  ⑤ Studies whose literatures are abstract, animal research and cadaver research.

### 2.4 Outcome indicator

(1) Primary outcome indicators: overall response rate, pain score (such as the visual analogue scale [VAS], etc);
(2) Secondary outcome indicators: joint function score (such as Lysholm score, Western Ontario and McMaster Universities Arthritis Index [WOMAC], and other scoring systems), daily life quality score (such as SF-36 score [the MOS item short from health survey], etc), and adverse reactions.

### 2.5 Search Strategy

The 2 researchers searched independently through search databases, including CNKI, Wanfang Data Knowledge Service Platform, VIP Information Chinese Journal Service Platform (VIP), China Biomedical Database, PubMed, EMBASE, Web of Science, and the Cochrane Library. The retrieval time was from the establishment of the database to March 2021. Chinese keywords

“knee osteoarthritis” (xi guan jie yan), “degenerative knee arthritis” (tui xing xing xi guan jie yan), “physical therapy” (wu li liao fa), “physical factor therapy” (wu li yin zi liao fa), “muscle strength training” (ji li xun lian), “electrotherapy” (dian liao), “pulse electromagnetic field” (mai chong dian ci chang), “extracorporeal shock wave” (ti wai chong ji bo), “whole body vibration” (quan shen zhen dong liao fa), “transcutaneous electrical nerve stimulation (TENS)” (jing pi shen jing dian ci ji), “middle frequency electrotherapy” (zhong pin dian liao), “high frequency electrotherapy” (gao pin dian liao); English keywords

“knee osteoarthritis,” “senile osteoarthritis,” “physiotherapy,” “modality,” “TENS,” etc. The 2 researchers selected the included literature independently according to the eligibility criteria. In case of any disagreement, the decision was made after consultation with the third researcher. PubMed retrieval strategies are shown in Table 1.

**Table 1:** Search strategy in PubMed database.

Number	Search terms
#1	Physiotherapy [Title/Abstract]
#2	Physical therapy [Title/Abstract]
#3	Modality [Title/Abstract]
#4	Physical factor therapy [Title/Abstract]
#5	Muscle strength training [Title/Abstract]
#6	Manual Therapy [Title/Abstract]
#7	Resistance exercise [Title/Abstract]
#8	Electrotherapy [Title/Abstract]
#9	Laser therapy [Title/Abstract]
#10	Pulse electromagnetic field [Title/Abstract]
#11	Ultrasound [Title/Abstract]
#12	Extracorporeal shock wave [Title/Abstract]
#13	Whole body vibration [Title/Abstract]
#14	Transcutaneous electrical nerve stimulation [Title/Abstract]
#15	TENS [Title/Abstract]
#16	Middle frequency electrotherapy [Title/Abstract]
#17	High frequency electrotherapy [Title/Abstract]
#18	Yoga [Title/Abstract]
#19	Aerobic exercise [Title/Abstract]
#20	Mind-body exercise [Title/Abstract]
#21	Aquatic exercise [Title/Abstract]
#22	#1 OR #2 OR #3 OR #4 OR #5 OR #6 OR #7 OR #8 OR #9 OR #10 OR #11 OR #12 OR #13 OR #14 OR #15 OR #16 OR #17 OR #18 OR #19 OR #20 OR #21
#23	knee osteoarthritis [MeSH]
#24	Knee Osteoarthritides [Title/Abstract]
#25	Osteoarthritis of Knee [Title/Abstract]
#26	Senile osteoarthritis [Title/Abstract]
#27	Knee arthritis [Title/Abstract]
#28	#23 OR #24 OR #25 OR #26 OR #27
#29	#22 AND #29

### 2.6 Data screening and extraction

**Figure 1:**
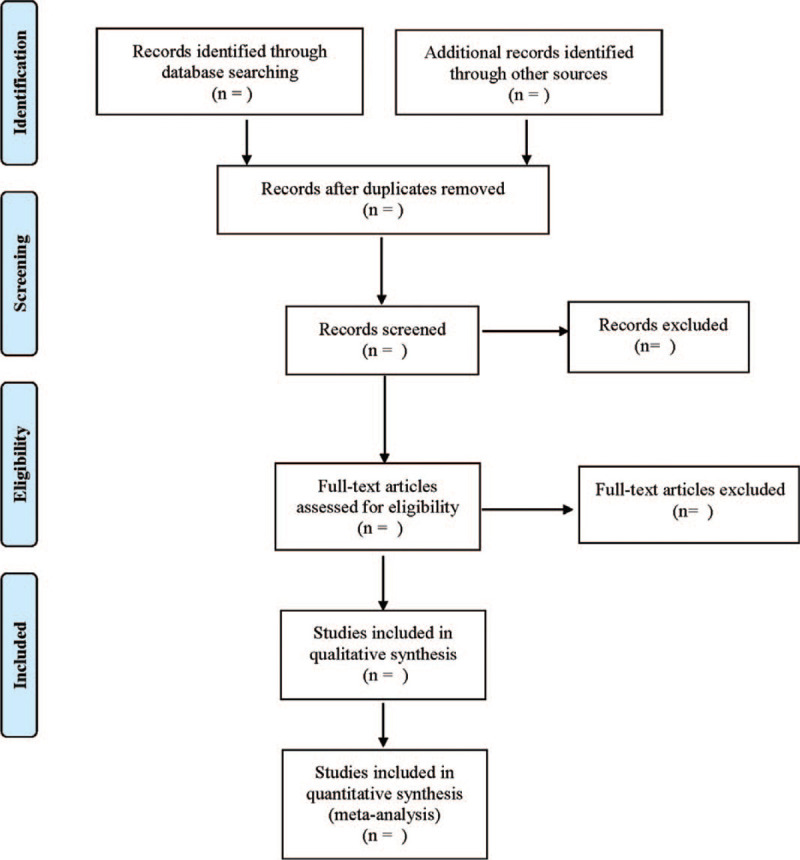
Flow diagram.

Literature screening and data extraction were conducted independently and cross-checked by 2 researchers. If there was any disagreement, discussion and decision were made with a third researcher. Relevant information was extracted as follows: first author, year of publication, KOA diagnostic criteria, sample size, sex, age, course of disease, study type, intervention, course of treatment, outcome indicators. The literature screening process is shown in Fig. 1.

### 2.7 Literature quality assessment

The quality evaluation of the literature was independently completed by 2 researchers according to the Cochrane Handbook for Systematic Reviews. In case of any disagreement, the third researcher participated in the discussion and finally determined the overall quality of the literature. Evaluation indicators included random sequence generation, allocation concealment, blind method, integrity of outcome data, selective reporting of study results, and other sources of bias. According to these indicators, the included literatures were evaluated as “high risk of bias,” “low risk of bias,” and “unknown.”

### 2.8 Statistical analysis

Stata 14.0 software was used to draw an evidence network map to show the comparison of the intervention measures for each outcome indicator. GEMTC14.3 based on the Bayesian framework was used for network meta-analysis. The effect values of binary variable were represented by odd ratio (OR), the effect values of continuous variables were represented by mean different (MD), and the results of statistical analysis were represented by 95% confidence interval (CI). The Markov Chain Monte Carlo (MCMC) fitting consistent model was used to carry out Bayesian inference. Four chains were used for simulation, the number of iterations was set as 50,000 (the first 20,000 were used for annealing and the last 30,000 were used for sampling), and the potential scale reduction factor (PSRF) was used to reflect the convergence degree of the model. When PSRF is close to or equal to 1, it indicates that data convergence is good and the obtained results are highly reliable.

### 2.9 Assessment of inconsistency

The inconsistency test should be carried out when there is a closed loop among the intervention measures, and the Z test performed by Stata 14.0 was used to evaluate the consistency between direct and indirect comparisons. If *P* ≥ .05, it means that there is less likely to be inconsistent between direct and indirect comparisons; if *P* < .05, it means that there is more likely to be inconsistent between direct and indirect comparisons, and fitting inconsistency analysis will be needed. Calculate the surface under the cumulative ranking curve (SUCRA) of different interventions by Stata 14.0. The larger the SUCRA value is, the better the efficacy of the intervention is. Finally, a comparison-correction diagram should be drawn to evaluate the existence of small sample effect.

### 2.10 Sensitivity analysis

In view of the fact that studies with different methodological quality levels may affect the final results, we will conduct sensitivity analysis by excluding studies with a high risk of bias.

### 2.11 Assessment of publication bias

If the number of studies is sufficient (n ≥ 10), funnel plots will be used to assess publication bias for included studies.^[15]^ If there is a difference in symmetry or distribution, it indicates that there is publication bias or small sample effect.

### 2.12 Evidence quality evaluation

We will use the Grading of Recommendation Assessment, Development and Evaluation (GRADE) scoring method to grade the evidence of the outcome index.^[16]^ And the quality of evidence will be rated as high, medium, low, or very low.

## 3 Discussion

Knee osteoarthritis is one of the most common chronic diseases and arthritis in the world, which is characterized by long course and easy recurrence, especially in elderly patients.^[17]^ Long-term and repeated chronic pain of knee joint and limitation of joint motion are the main factors affecting the daily life of patients with knee osteoarthritis. The aim of knee osteoarthritis treatment is to relieve clinical symptoms, improve joint function, delay the progression of the disease, and reduce the degree of disability associated with it as much as possible.^[18]^ Non-Steroidal Anti-inflammatory Drugs (NSAIDs) are the main suggestion for conservative treatment recommended by relevant clinical guidelines. However, adverse reactions of NSAIDs have seriously affected patients’ compliance with the drugs and inevitably reduced the therapeutic effect. Non-propulsive physical therapy has high safety and long-lasting efficacy, which plays an important role in conservative treatment regimens^[19]^ and has been recommended in ACR guidelines.^[20]^ There are many physical therapy schemes, and clinical studies show that the efficacy of each scheme is not the same. For example, muscle strength training can enhance lower limb muscle strength, relieve pain, and improve quality of life in patients with KOA. The effect of pain improvement is better than that of aerobic exercise^[21]^; ultrasonic therapy can significantly improve pain and enhance the range of motion of joints^[22]^; whole body vibration therapy plays a positive role in improving joint function, but has little effect on pain.^[23]^ As there is no direct comparison between the treatments, it is impossible to objectively evaluate the differences in the efficacy of different schemes, which is inconvenient for clinical selection of appropriate treatment schemes. Therefore, this study will explore the differences in the efficacy of different physical therapy in the treatment of KOA by means of network meta-analysis, so as to provide evidence-based basis for clinical decision-makers to choose the best scheme. However, there are some limitations in our study. Due to the limitation of language retrieval, we will only include Chinese and English literatures, which may cause selection bias. Factors such as KOA stage, physical therapy mode, and treatment duration may increase the possibility of heterogeneity. Nevertheless, we believe that the results of this study will help determine the best physiotherapy regimen for the treatment of KOA.

## Author contributions

**Data curation:** Anju Xiao, Zongguang Tian.

**Data collection:** Zongguang Tian, Anju Xiao.

**Funding acquisition:** Daoming Xu, Anju Xiao.

**Investigation:** Weisen Cai, Daoming Xu.

**Literature retrieval:** Weisen Cai, Daoming Xu.

**Software:** Anju Xiao, Zongguang Tian.

**Supervision:** Tong Wang.

**Writing – original draft:** Weisen Cai, Daoming Xu.

**Writing – review & editing:** Zongguang Tian, Tong Wang.
